# Knowledge, attitudes, and practices of pregnant women regarding fetal growth restriction: a cross-sectional study

**DOI:** 10.3389/fpubh.2025.1567038

**Published:** 2025-08-07

**Authors:** Yanfei Zhu, Chenyang Ding, Lili Fang, Zhuchun Mei, Xiaoe Xie

**Affiliations:** Obstetrical Department of Lishui Maternal and Child Health Hospital, Lishui, China

**Keywords:** fetal growth restriction, health knowledge, attitudes, practices, pregnant women, cross-sectional study, structural equation modeling

## Abstract

**Objective:**

To investigate the knowledge, attitudes, and practices (KAP) of pregnant women regarding fetal growth restriction (FGR).

**Methods:**

This cross-sectional study was conducted from December 2024 to January 2025 in Lishui City, Zhejiang Province, with pregnant women as study participants. A self-administered questionnaire was used to collect demographic information and assess KAP scores.

**Results:**

A total of 381 participants were included in the study. The majority were <30 years old (55.12%), pregnant for >32 weeks (56.17%), and with their first pregnancy (48.56%). The mean scores for knowledge, attitudes, and practices were 11.08 ± 5.44 (out of 22, 50.36%), 30.33 ± 3.07 (out of 40, 75.83%), and 37.72 ± 2.99 (out of 40, 94.30%), respectively. Knowledge scores were positively correlated with attitude (*r* = 0.1269, *p* = 0.0132) and practice (*r* = 0.2838, *p* < 0.001) scores. The attitude scores were correlated with the practice scores (*r* = 0.2140, *p* < 0.001). Structural equation modeling (SEM) revealed that knowledge had direct influences on attitudes (*β* = 0.17, *p* < 0.001) and practices (*β* = 0.11, *p* = 0.004). Attitudes had a direct influence on practices (*β* = 0.61, *p* < 0.001). Knowledge had an indirect influence on practices through attitudes (*β* = 0.10, *p* < 0.001).

**Conclusion:**

Pregnant women exhibited limited knowledge but demonstrated favorable attitudes and proactive practices regarding FGR. Improving the knowledge about FGR through educational interventions could enhance both attitudes and practices.

## Introduction

Fetal growth restriction (FGR) (also called intrauterine growth restriction [IUGR]) refers to an ultrasound-estimated fetal weight or abdominal circumference <10th percentile for gestational age (1–3). FGR is relatively common and reported to occur in about 10% of pregnancies (2). The risk factors for FGR include maternal factors (such as prior pregnancy with FGR, hypertension, smoking, medication, malnutrition, or maternal infection), fetal factors (such as congenital malformations, aneuploidy, or multiple gestation), and placental factors (such as placental mosaicism, placenta previa, chronic abruption, infarction, or velamentous cord insertion) (1–3). The complications may include preeclampsia, preterm birth, low birth weight, intrauterine fetal demise, respiratory distress syndrome, sepsis, and perinatal mortality (1–3). Therefore, early diagnosis and management of FGR are critical for maternal and fetal health. Monitoring should be initiated as soon as FGR is diagnosed, including serial ultrasound estimation of fetal weight every 2 weeks, umbilical artery Doppler, placental assessment, and other Doppler studies, such as middle cerebral artery (MCA), umbilical vein (UV), and ductus venosus (DV) (2, 3). Besides fetal monitoring, the management of FGR involves determining the best timing for delivery. Severe FGR is considered an indication of the induction of labor (1). If the underlying etiology is known, estimated gestational age and other clinical findings during antenatal fetal surveillance should inform decisions (1).

Although prenatal care can help identify pregnancies at high risk of FGR, early intervention measures still depend on pregnant women’s awareness, attitudes, and practices regarding FGR. Pregnant women also need to be aware of the importance of ultrasound screening and of the possible adverse consequences of missing an FGR diagnosis for the fetus. Indeed, screening and managing FGR require strictly adhering to the physician’s advice and management plan. Knowledge, attitude, and practice (KAP) studies enable the investigation of gaps, misunderstandings, and misconceptions that constitute barriers to the optimal performance of a given health-related action or topic in a specific population (4, 5). KAP studies are particularly helpful for preparing and designing educational and motivational interventions to improve a specific aspect of healthcare. Currently, there is very limited data on the KAP of pregnant women toward FGR. One study performed in Washington, D. C., showed that most of the 150 participants believed that prescription drugs could lead to FGR (6). Another study suggested that pregnancy health education could help reduce the occurrence of FGR (7). Two studies reported the misconception that exercise during pregnancy can lead to FGR (8, 9). The knowledge and attitudes of pregnant women about FGR prevention and management may directly influence their health behaviors and lifestyles, thereby impacting fetal health outcomes.

Therefore, this study aimed to investigate the KAP of pregnant women regarding FGR.

## Methods

### Study design and participants

This cross-sectional study was conducted from December 6, 2024, to January 31, 2025 (a total of 57 days), in Lishui City, Zhejiang Province, and involved pregnant women as participants. The inclusion criteria were (1) age ≥ 18 years, (2) confirmed pregnancy by ultrasound examination, and (3) voluntary participation with signed informed consent. The questionnaires completed in <90 s or with identical responses for all items (e.g., selecting only the first option for all items) were deemed invalid. The study was approved by the Ethics Committee of Lishui Maternal and Child Health Hospital (approval number: 2411121811859). Informed consent was obtained from all participants, and the study adhered to the principles outlined in the Declaration of Helsinki.

### Questionnaire

The initial draft of the questionnaire was designed by the investigators based on *Guideline No. 442: Fetal Growth Restriction: Screening, Diagnosis, and Management in Singleton Pregnancies (2023)* (10). A preliminary survey was conducted to refine the second draft and assess face validity. Twenty-six questionnaires were retrieved. The questionnaire’s overall Cronbach’s *α* coefficient was 0.883, indicating acceptable internal consistency.

The final questionnaire, which was in Chinese, consisted of four sections: demographic data, knowledge dimension, attitude dimension, and practice dimension. The demographic section collected data on age, gestational week, place of residence, education level, type of work, average monthly income per capita, marital status, number of previous pregnancies, and number of previous deliveries. The knowledge dimension consisted of 12 items. Item K3 was a multiple-choice question used only for descriptive statistics and not scored. The remaining 11 items were scored 2, 1, or 0, indicating well-understood, of, or unclear, respectively, with a total score ranging from 0 to 22. The attitude dimension contained eight items. Items A2, A6, A7, and A8 were positively scored (strongly agree to strongly disagree, 5 to 1 points). The remaining items were reverse-scored (strongly disagree to strongly agree, 1 to 5 points), yielding a total score of 8–40. The practice dimension consisted of eight items, all of which were scored positively (always to never, 5 points to 1 point), with a total score ranging from 8 to 40. Good knowledge, positive attitudes, and proactive practices were defined as scores above 60% of the total score (11, 12).

### Questionnaire distribution and quality control

The questionnaire was uploaded to the Questionnaire Star platform to generate a QR code. Participants were invited to participate during prenatal examinations. The QR code was shared via a WeChat group or scanned in person. The first page of the questionnaire contained the informed consent form. Participation in the study required consenting to participate.

Each IP address was restricted to a single submission. Responses to all questions were mandatory for submission. To prevent multiple entries from the same participant, the IP addresses used to submit the questionnaire were retained during the data collection period but subsequently deleted from the database. Participants were assured that their data would be anonymous and could not be used to identify them. The data was securely stored on a server at the study hospital, accessible only to the research team.

### Sample size calculation

The minimum sample size was determined using the method for quantitative surveys (13), i.e., requiring at least 10 times the number of survey items. With 38 items in the questionnaire, the minimum sample size was calculated to be 380.

### Statistical analysis

Statistical analysis was performed using STATA 17 (StataCorp LP, College Station, TX, United States). Continuous variables were tested for normal distribution using the Kolmogorov–Smirnov test. Normally distributed continuous variables were expressed as mean ± standard deviation and analyzed using Student’s t-test (for two groups) or ANOVA (for three or more groups). Non-normally distributed variables were expressed as median (interquartile range [IQR]) and analyzed using the Mann–Whitney U test (for two groups) or the Kruskal-Wallis H-test (for three or more groups). Categorical data were expressed as n (%) and analyzed using the chi-squared test or Fisher’s exact test. Spearman correlation was used to analyze relationships among knowledge, attitude, and practice scores. Structural equation modeling (SEM) was conducted to test the following hypotheses: (H1) knowledge directly affects attitudes, (H2) knowledge directly affects practices, and (H3) knowledge indirectly affects practices through attitudes. Model fit was evaluated using root mean square error of approximation (RMSEA), standardized root mean square residual (SRMR), Tucker-Lewis index (TLI), and comparative fit index (CFI). Two-sided *p*-values <0.05 were considered statistically significant.

## Results

### Characteristics of the participants

A total of 411 participants were invited to participate. Twelve participants declined to participate, and 399 completed the questionnaire. Three questionnaires were completed in <90 s, four participants reported being under 18 years old, and 12 had logical errors (e.g., reporting no previous pregnancy but indicating a delivery). Therefore, 381 valid questionnaires were analyzed.

The majority of the participants were under 30 years old (55.12%), pregnant for 32 weeks or more (56.17%), living in urban areas (54.59%), had a bachelor’s degree or above (40.68%), had a long-term stable job (54.59%), had a monthly per capita income of 5,000–9,999 CNY (47.77%), were in their first pregnancy (48.56%), and had not previously delivered (60.37%; Table 1).

**Table 1:** Characteristics of the participants.

*n* = 381	n (%)	Knowledge score		Attitude score		Practice score	
		Mean±SD	*p*	Mean±SD	*p*	Mean±SD	*p*
Total score		11.08 ± 5.44		30.33 ± 3.07		37.72 ± 2.99	
Age (years) [range: 18–43]	29.39 ± 4.40		0.516		0.786		0.240
<30	210 (55.12)	11.25 ± 5.53		30.28 ± 3.07		37.82 ± 3.06	
≥30	171 (44.88)	10.86 ± 5.33		30.38 ± 3.07		37.59 ± 2.89	
Gestational week (weeks)			0.535		0.340		0.526
12 or less	19 (4.99)	10.36 ± 4.49		29.84 ± 2.21		37.05 ± 3.27	
13–23	66 (17.32)	10.48 ± 5.44		30.69 ± 2.99		37.93 ± 2.68	
24–27	41 (10.76)	10.29 ± 5.95		31.19 ± 3.57		38.07 ± 2.43	
28–31	41 (10.76)	10.80 ± 5.78		30.14 ± 3.44		36.95 ± 3.76	
32 or more	214 (56.17)	11.52 ± 5.35		30.12 ± 2.96		37.79 ± 2.97	
Place of residence			0.308		0.061		0.618
Rural	126 (33.07)	10.47 ± 4.97		29.95 ± 2.99		37.33 ± 3.56	
Urban	208 (54.59)	11.35 ± 5.77		30.63 ± 3.17		37.84 ± 2.74	
Suburban	47 (12.34)	11.46 ± 5.11		29.93 ± 2.69		38.21 ± 2.15	
Education level			0.247		**<0.001**		**0.035**
Middle school or below	50 (13.12)	11.62 ± 5.32		28.68 ± 2.13		37.1 ± 3.63	
High school/technical school	63 (16.54)	10.33 ± 5.29		29.74 ± 2.63		38.07 ± 2.57	
Associate degree	113 (29.66)	10.83 ± 5.57		30.27 ± 3.23		37.33 ± 2.94	
Bachelor’s degree or above	155 (40.68)	11.38 ± 5.45		31.12 ± 3.12		38.05 ± 2.90	
Type of work			0.104		**0.003**		0.092
Long-term stable job (fixed employment)	208 (54.59)	11.21 ± 5.45		30.76 ± 2.99		37.93 ± 2.93	
Freelancer	54 (14.17)	11.94 ± 5.28		30.03 ± 2.80		38.05 ± 2.63	
Unemployed	40 (10.5)	9.2 ± 4.45		30.22 ± 3.63		36.72 ± 3.19	
Homemaker	34 (8.92)	10.29 ± 6.00		29.35 ± 2.38		37.47 ± 3.71	
Other	45 (11.81)	11.68 ± 5.73		29.44 ± 3.34		37.4 ± 2.72	
Average monthly income per capita (CNY)			0.068		0.096		**0.043**
<5,000	120 (31.5)	10.24 ± 5.09		29.9 ± 3.05		37.31 ± 3.17	
5,000–9,999	182 (47.77)	11.58 ± 5.65		30.42 ± 3.03		37.79 ± 2.81	
≥10,000	79 (20.73)	11.18 ± 5.36		30.73 ± 3.13		38.17 ± 3.04	
Marital status			0.899		0.412		0.823
Single	20 (5.25)	11 ± 4.55		29.7 ± 2.38		37.45 ± 3.03	
Married	361 (94.75)	11.08 ± 5.49		30.36 ± 3.10		37.73 ± 2.98	
Number of previous pregnancies			0.111		0.236		0.557
0	185 (48.56)	10.82 ± 5.39		30.50 ± 2.99		37.76 ± 3.19	
1	104 (27.3)	11.85 ± 5.08		30.23 ± 3.11		37.66 ± 2.77	
≥2	92 (24.15)	10.70 ± 5.90		30.06 ± 3.18		37.69 ± 2.81	
Number of previous deliveries			0.611		0.411		0.342
0	230 (60.37)	11.00 ± 5.27		30.43 ± 2.99		37.77 ± 3.11	
1	127 (33.33)	11.00 ± 5.68		30.13 ± 3.24		37.70 ± 2.82	
≥2	24 (6.3)	12.12 ± 5.92		30.33 ± 2.98		37.33 ± 2.69	

Bold values represents is that *p* is less than 0.05.

### Knowledge

The mean knowledge score was 11.08 ± 5.44 out of a maximum of 22 (50.36%), indicating poor knowledge of FGR. No demographic factors were associated with the knowledge scores (Table 1). The knowledge item with the lowest score was K1 (8.66% very familiar; “FGR refers to a condition in which the fetus fails to reach its expected growth potential during pregnancy, primarily characterized by estimated fetal weight or abdominal circumference below the 10th percentile for gestational age”), while the item with the highest score was K9 (37.27% very familiar; “Singleton pregnant women diagnosed with FGR are advised to count fetal movements daily. If fetal movements decrease, they should seek medical attention promptly”) (Table 2). The reported sources of knowledge on FGR (multiple choices allowed) were medical books and materials (*n* = 161, 42.26%), hospital lectures and education (*n* = 123, 32.28%), new media (e.g., WeChat, Weibo; *n* = 280, 73.49%), multimedia (e.g., television) (n = 137, 35.96%), and relatives and friends (*n* = 104, 27.3%; Table 2).

**Table 2:** Knowledge distribution.

Items, n (%)	Very familiar	Heard of it	Unclear
1. Fetal growth restriction (FGR) refers to a condition in which the fetus fails to reach its expected growth potential during pregnancy, primarily characterized by estimated fetal weight or abdominal circumference below the 10th percentile for gestational age.	33 (8.66)	190 (49.87)	158 (41.47)
2. Early and timely diagnosis of FGR can effectively reduce fetal mortality.	61 (16.01)	232 (60.89)	88 (23.1)
3. Causes of FGR include (Multiple choices):			
a. Maternal factors: maternal nutritional status, pregnancy complications (e.g., chronic hypertension, diabetes)	361 (94.75)		
b. Fetal factors: chromosomal abnormalities, structural anomalies, intrauterine infections	346 (90.81)		
c. Placental abnormalities: morphological abnormalities, infarction, tumors	307 (80.58)		
d. Umbilical cord abnormalities: single umbilical artery, thin umbilical cord, cord torsion, cord knot	314 (82.41)		
4. Screening methods for FGR include medical history collection, fundal height measurement, ultrasound examination, and blood tests.	62 (16.27)	242 (63.52)	77 (20.21)
5. Pregnant women suspected of FGR should undergo ultrasound as early as possible to further evaluate fetal growth indicators.	96 (25.2)	231 (60.63)	54 (14.17)
6. Accurate gestational age verification is a crucial prerequisite for diagnosing FGR.	102 (26.77)	227 (59.58)	52 (13.65)
7. Supplementing energy and protein may improve fetal growth and reduce the risk of FGR.	92 (24.15)	228 (59.84)	61 (16.01)
8. Pregnant women with chronic diseases should reduce the impact of these conditions and medications on the fetus under the guidance of specialists to lower the risk of FGR.	83 (21.78)	229 (60.1)	69 (18.11)
9. Singleton pregnant women diagnosed with FGR are advised to count fetal movements daily. If fetal movements decrease, they should seek medical attention promptly.	142 (37.27)	195 (51.18)	44 (11.55)
10. Pregnant women diagnosed with FGR need a comprehensive assessment of maternal and fetal conditions to decide the mode of delivery (vaginal delivery or cesarean section).	107 (28.08)	208 (54.59)	66 (17.32)
11. For FGR fetuses above 28 weeks of gestation, if monitoring is normal, full-term delivery can be considered.	70 (18.37)	207 (54.33)	104 (27.3)
12. During labor, FGR fetuses are more prone to intrapartum distress.	62 (16.27)	212 (55.64)	107 (28.08)

### Attitudes

The mean attitude score was 30.33 ± 3.07 out of a maximum of 40 (75.83%), indicating favorable attitudes toward FGR. The attitude scores were associated with education level (*p* < 0.001) and type of work (*p* = 0.003; Table 1). The attitude item with the lowest score was A3 (reverse scored; 84.51% strongly agree and agree; “I am concerned about experiencing FGR myself”), while the item with the highest score was A7 (98.69% strongly agree and agree; “I think babies with FGR should undergo continuous neurodevelopmental assessments after birth”; Table 3).

**Table 3:** Attitude distribution.

Items, n (%)	Strongly agree	Agree	Neutral	Disagree	Strongly disagree
1. I believe FGR is not a severe condition and does not need much attention.	5 (1.31)	9 (2.36)	6 (1.57)	180 (47.24)	181 (47.51)
2. I think pregnant women should pay attention to fetal growth to identify FGR promptly.	256 (67.19)	117 (30.71)	1 (0.26)	3 (0.79)	4 (1.05)
3. I am concerned about experiencing FGR myself.	111 (29.13)	211 (55.38)	4 (1.05)	36 (9.45)	19 (4.99)
4. I believe that once FGR is diagnosed, early delivery is the only option.	39 (10.24)	162 (42.52)	6 (1.57)	158 (41.47)	16 (4.2)
5. I believe FGR pregnancies can only be resolved via cesarean delivery.	23 (6.04)	144 (37.8)	17 (4.46)	172 (45.14)	25 (6.56)
6. I believe FGR mothers are prone to psychological problems postpartum and should receive psychological support.	174 (45.67)	186 (48.82)	8 (2.1)	11 (2.89)	2 (0.52)
7. I think babies with FGR should undergo continuous neurodevelopmental assessments after birth.	166 (43.57)	210 (55.12)	2 (0.52)	2 (0.52)	1 (0.26)
8. I believe pregnant women and their families should actively learn about FGR.	215 (56.43)	161 (42.26)	4 (1.05)	/	1 (0.26)

### Practices

The mean practice score was 37.72 ± 2.99 out of a maximum of 40 (94.30%), indicating proactive practices toward FGR. The practice scores were associated with education level (*p* = 0.035) and average monthly income (*p* = 0.043; Table 1). The practice item with the lowest score was P4 (83.73%; “If FGR is diagnosed, I will stay positive.”), while the item with the highest score was P5.1 (98.69%; “To prevent FGR, I will avoid smoking and drinking alcohol”; Table 4).

**Table 4:** Practice distribution.

Items, n (%)	Strongly agree	Agree	Neutral	Disagree	Strongly disagree
I will undergo regular prenatal checkups to evaluate fetal growth.	321 (84.25)	53 (13.91)	6 (1.57)	1 (0.26)	/
If a prenatal checkup shows poor fetal growth, I will actively ask healthcare providers whether FGR is present.	277 (72.7)	87 (22.83)	14 (3.67)	2 (0.52)	1 (0.26)
If FGR is diagnosed, I will follow the doctor’s advice on the mode and timing of delivery.	299 (78.48)	76 (19.95)	5 (1.31)	1 (0.26)	/
If FGR is diagnosed, I will maintain an optimistic attitude.	202 (53.02)	117 (30.71)	57 (14.96)	4 (1.05)	1 (0.26)
To prevent FGR:					
5.1 I will avoid smoking and drinking alcohol.	333 (87.4)	43 (11.29)	4 (1.05)	1 (0.26)	/
5.2 I will avoid taking medications that may affect fetal growth.	323 (84.78)	48 (12.6)	9 (2.36)	1 (0.26)	/
5.3 I will strictly adhere to the doctor’s recommended weight gain targets during pregnancy.	279 (73.23)	94 (24.67)	7 (1.84)	1 (0.26)	/
5.4 I will appropriately supplement energy and protein.	288 (75.59)	84 (22.05)	7 (1.84)	2 (0.52)	/

### Correlations

The knowledge scores were correlated with attitudes (*r* = 0.1269, *p* = 0.0132) and practices (*r* = 0.2838, *p* < 0.001) scores. The attitude scores were correlated with the practice scores (*r* = 0.2140, *p* < 0.001; Supplementary Table S1).

### SEM

**Figure 1:**
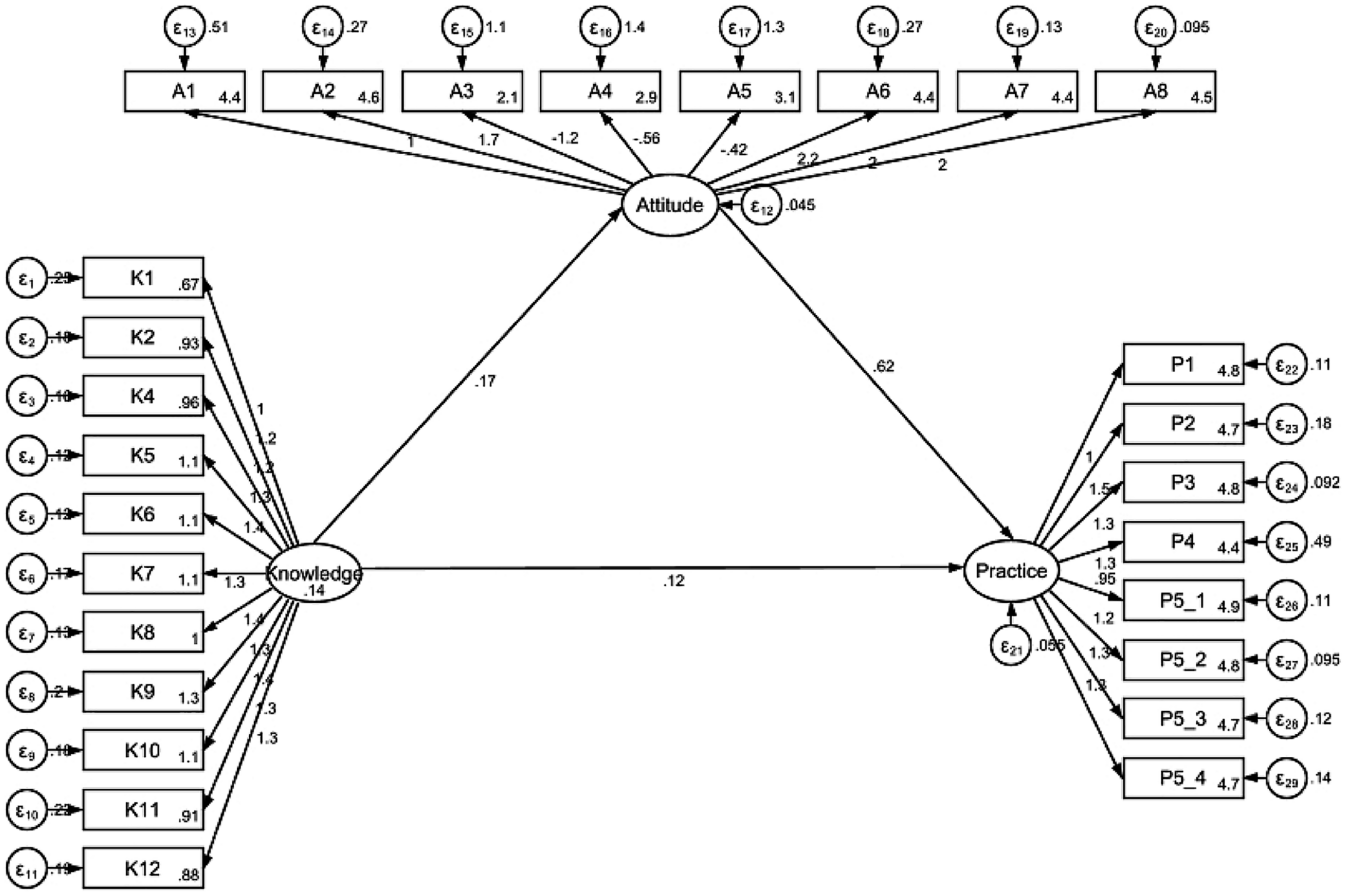
Structural equation model analysis.

The SEM analysis (Figure 1) was stable, with all fit indexes meeting the criteria for analysis validity (Supplementary Table S2). Knowledge had a direct influence on attitudes (*β* = 0.17, *p* < 0.001) and practices (*β* = 0.11, *p* = 0.004). Attitudes had a direct influence on practices (*β* = 0.61, *p* < 0.001). Knowledge had an indirect influence on practices through attitudes (*β* = 0.10, *p* < 0.001; Table 5).

**Table 5:** Structural equation modeling analysis for direct and indirect effects.

Model paths	Total effects		Direct effect		Indirect effect	
	β (95% CI)	*p*	β (95% CI)	*p*	β (95% CI)	*p*
Asum ←						
Ksum	0.17 (0.08,0.26)	<0.001	0.17 (0.08,0.26)	<0.001		
Psum ←						
Asum	0.61 (0.35,0.87)	<0.001	0.61 (0.35,0.87)	<0.001		
Ksum	0.22 (0.13,0.31)	<0.001	0.11 (0.03,0.19)	0.004	0.10 (0.05,0.15)	<0.001

CI, confidence interval.

## Discussion

This cross-sectional study investigated the KAP of pregnant women regarding FGR. The results suggested that pregnant women have poor knowledge but favorable attitudes and proactive practices toward FGR. Most knowledge items displayed poor scores. Knowledge of FGR should be improved through educational interventions, which could lead to improvements in attitudes and practices.

No previous studies have focused specifically on KAP toward FGR; however, some scarce data are available in the literature. Indeed, Kazma et al. (6) performed a study on 150 American women living in Washington, D. C. and reported that the majority of their participants believed that prescription drugs could cause FGR. Two other studies, one from Spain (8) and the other from Nigeria (9), reported that many women believed physical exercise during pregnancy could lead to FGR. Although it is true that some prescription drugs can be related to FGR, not all prescription drugs cause FGR (1–3). On the other hand, physical activity during pregnancy is not associated with FGR (14), but many women become fearful of physical activity once they receive an FGR diagnosis for their fetuses (15). In the present study, knowledge of FGR was limited, but attitudes and practices were favorable, suggesting that many women were following the advice of healthcare providers without fully understanding the underlying reasons.

In the present study, the KAP dimension scores were correlated with each other. Additionally, the SEM analysis indicated that knowledge had a significant influence on attitudes and practices. Hence, improving knowledge of FGR should translate into more positive attitudes and more active practices. Indeed, according to the KAP theory, knowledge serves as the basis for practices, while attitudes are the driving force behind these practices (4, 5). For instance, Wen et al. (7) showed that educational interventions about health during pregnancy could reduce the occurrence of FGR. In the present study, nearly all knowledge items displayed poor knowledge, including the definition and signs of FGR, the importance of early diagnosis, screening methods, the role of ultrasound follow-up, the necessity of accurate gestational age determination, the management of FGR, the influence of comorbidities, self-monitoring of fetal health, the importance of discussing delivery modalities, and the impact of FGR on the risk of labor complications. Educational interventions should be designed and tested in future studies to further enhance their effectiveness. Such interventions could take various forms, including pamphlets, websites, short videos, and educational sessions led by nurses. Although no previous studies have examined interventions to improve knowledge of FGR, it is well established from various medical topics that such interventions can enhance knowledge of a topic, ultimately leading to better practices (16, 17). Of course, prenatal education is well-known for improving knowledge about various topics pertaining to pregnancy and the postpartum period (18–20). Since many patients consider healthcare providers a reliable source of health-related information (21), future studies could examine the KAP of healthcare providers toward FGR. Educational interventions can also be designed to improve the KAP of healthcare providers, increasing their ability to disseminate correct information (22).

The knowledge scores were poor, but the attitude and practice scores were acceptable. Nevertheless, some attitude and practice items would need improvement. Indeed, a substantial proportion of women were concerned about having a fetus with FGR. These women should be reassured through proper management and monitoring. Although there are some possible complications, most are preventable or manageable if taken early. More than half of the women believed that early delivery was the only option for managing FGR, and many assumed that cesarean delivery was the only viable method. Patients should be reassured that while early delivery is one possible approach, it is not the sole option. Decisions regarding delivery will be carefully made after evaluating all relevant factors (1). Additionally, the practice item with the lowest score was related to maintaining a positive attitude following an FGR diagnosis, underscoring the need for adequate psychological and social support to help women cope with such a diagnosis.

In the present study, no factors were associated with the knowledge scores. However, higher education levels were associated with higher attitude and practice scores. Stable long-term work was associated with higher attitude scores, and higher income was associated with higher practice scores. A better socioeconomic status has been associated with better healthcare literacy (23). In China, a better socioeconomic status is also associated with easier access to healthcare resources (24). Nevertheless, the results suggest that women with lower socioeconomic status should be prioritized for educational and motivational interventions about FGR.

This study has several limitations. First, it was a cross-sectional study, which prevents the determination of causality. A SEM analysis was performed as a surrogate for causality to examine the relationships among KAP dimensions, but the results should be interpreted with caution, as causality in SEM is statistically inferred rather than directly observed (13, 25, 26). Additionally, cross-sectional studies provide only a snapshot of a situation at a specific point in time. Nevertheless, these results could serve as a historical baseline for future studies. Second, the participants were all from the same city, which limits the generalizability of the results. The questionnaire was designed by the investigators and may reflect local practices, customs, and policies, which further restricts its generalizability and applicability. Third, while the study aimed to assess the general KAP of pregnant women regarding FGR, some participants may have been diagnosed with FGR, as we did not specifically identify or track such cases. This lack of differentiation between women with FGR and those without may have influenced the results. In future studies, it would be valuable to collect medical records or use other methods to identify women with FGR and compare their responses to those of women without FGR. Finally, all KAP studies are subject to social desirability bias (27, 28). However, given the low knowledge scores and high attitude and practice scores, this bias is unlikely to have significantly influenced the findings.

In conclusion, pregnant women displayed poor knowledge but favorable attitudes and proactive practices toward FGR. Most knowledge items scored poorly. Knowledge of FGR should be improved through educational interventions, which could lead to better attitudes and practices. Future studies should focus on designing and testing such interventions and evaluating their impact on KAP and pregnancy outcomes.

## Data Availability

The original contributions presented in the study are included in the article/Supplementary material, further inquiries can be directed to the corresponding author.
