# Supplementary material for: Knowledge, attitudes, and practices of pregnant women regarding fetal growth restriction: a cross-sectional study
Source: Front Public Health. 2025 Aug 7;13:1567038. doi: 10.3389/fpubh.2025.1567038 (PMC12367642; doi:10.3389/fpubh.2025.1567038)
Supplement: Supplementary file 1 [file Table_1.docx]

**Supplementary Table S1.** Correlation analysis

|  | **Knowledge** | **Attitudes** | **Practices** |
| --- | --- | --- | --- |
| Knowledge | 1 |  |  |
| Attitudes | 0.1269 (P=0.0132) | 1 |  |
| Practices | 0.2838 (P<0.001) | 0.2140 (P<0.001) | 1 |

**Supplementary Table S2.** Model fit indices

| Indicators | Reference | Results |
| --- | --- | --- |
| RMSEA | <0.08 Good | 0.077 |
| SRMR | <0.08 Good | 0.061 |
| TLI | >0.8 Good | 0.844 |
| CFI | >0.8 Good | 0.857 |

RMSEA: root mean square error of approximation; SRMR: standardized root mean square residual; TLI: Tucker-Lewis Index; CFI: comparative fit index.
